# Role of interferon regulatory factor 7 in corneal endothelial cells after HSV-1 infection

**DOI:** 10.1038/s41598-021-95823-9

**Published:** 2021-08-13

**Authors:** Fumie Ohtani, Dai Miyazaki, Yumiko Shimizu, Tomoko Haruki, Satoru Yamagami, Yoshitsugu Inoue

**Affiliations:** 1 Department of Ophthalmology, Tottori University, Yonago, Tottori Japan; 2 Department of Ophthalmology, Nihon University School of Medicine, Tokyo, Japan

**Keywords:** Herpes virus, Molecular medicine, Infection, Corneal diseases, Infection

## Abstract

Viral infections of the cornea including herpes simplex virus 1 (HSV-1) cause visual morbidity, and the corneal endothelial cell damage leads to significant visual impairment. Interferon regulatory factor 7 (IRF7) has been identified as a significant regulator in corneal endothelial cells after an HSV-1 infection. To examine the role played by IRF7, the DNA binding domain (DBD) of IRF7 of human corneal endothelial cells (HCEn) was disrupted. An RNAi inhibition of IRF7 and IRF7 DBD disruption (IRF7 ∆DBD) led to an impairment of IFN-β production. Impaired IFN-β production by IRF7 ∆DBD was regained by IRF7 DNA transfection. Transcriptional network analysis indicated that IRF7 plays a role in antigen presentation function of corneal endothelial cells. When the antigen presentation activity of HCEn cells were examined for priming of memory CD8 T cells, IRF7 disruption abolished the anti-viral cytotoxic T lymphocyte (CTL) response which was dependent on the major histocompatibility complex (MHC) class I. To further examine the roles played by IRF7 in CTL induction as acquired immunity, the contribution of IRF7 to MHC class I-mediated antigen presentation was assessed. Analysis of IRF7 ∆DBD cells indicated that IRF7 played an unrecognized role in MHC class I induction, and the viral infection induced-MHC class I induction was abolished by IRF7 disruption. Collectively, the IRF7 in corneal endothelial cells not only contributed to type I IFN response, but also to the mediation of viral infection-induced MHC class I upregulation and priming of CD8 arm of acquired immunity.

## Introduction

A decrease of corneal clarity causes significant visual morbidity and generally requires corneal transplantation. Currently, the leading cause of a reduction of clarity is bullous keratopathy which results from a dysfunction of the corneal endothelial cells. This dysfunction develops by physiological aging, surgical procedures, or viral infections. Viral infections are manifested as keratitis or endotheliitis. Herpes simplex virus (HSV) and cytomegalovirus (CMV) are the most common pathogens to cause endotheliitis. These infections are important because these pathogens are ubiquitous, and HSV persists in the trigeminal ganglia innervating the cornea for long periods and can be reactivated. In addition, corneal latency has also been suggested from infections of host eyes by HSV positive donor corneas^1^. Therefore, prolonged treatment and management is required which causes a significant burden on the health care budget.

To develop efficacious drugs or management protocols to treat endotheliitis, it is necessary to understand how corneal endothelial cell react to these pathogens. We have analyzed how corneal endothelial cells respond to infection by HSV or CMV by examining corneal endothelial cells in culture^2–4^. Earlier, we analyzed the global transcriptional responses, and we reported that corneal endothelial cells produce characteristic interferon (IFN) responses as its major network^3,4^.

Generally, IFN responses are the canonical responses that protect the host from viral infections by the production of IFNs. IFNs are classified as type I, type II, and type III, and they have broad functions and are secreted by different cell populations^5^. For example, type I IFN, including IFN-α and -β, are secreted by many cell types including lymphocytes and endothelial cells.

These distinct IFN profiles are dictated by the IFN regulatory factor (*IRF*) and the signal transducer and activator of transcription (*STAT*) families, i.e., the IRF and STAT transcriptional factors. The IRF proteins are special pivotal transcription factors related to the induction of IFNs. The roles of the IRF proteins are not limited to IFN secretion, but they are also involved in the induction of the IFN stimulated gene (ISG) proteins which have strong anti-viral activity and act as another layer of innate immunity. The roles of IRF are diverse and not limited to IFN responses. The IRFs exert their diverse roles by the binding of their DNA binding domain (DBD) to the interferon-stimulated response element (*ISRE*) regions which are found on promoter sequences of a number of genes.

IRF proteins have distinctive roles and expression profiles depending on the cellular lineages. IRF3 and IRF7 are the best-known members of the anti-viral IRF family. Of these, IRF3 is considered important for non-immune cells because a number of cell populations that express IRF3 in the steady state, are upregulated by infection.

Based on the transcriptional network analysis of corneal endothelial cells after HSV-1 or CMV infection, we identified IRF7 as the most significant upstream regulator in the corneal endothelial cells. IRF7 was recognized earlier as specific to plasmacytoid dendritic cells (pDC) or antigen specific cells (APCs). However, corneal endothelial cells are not professional APCs, and it is not clear how IRF7 can contribute to the protection from viral infections.

Thus, the purpose of this study was to determine the roles played by IRF7 in anti-viral immune responses. To accomplish this, we first examined its roles in the induction of type I IFN, and then assessed its contribution to acquired immune responses. We shall show that the IRF7 in corneal endothelial cells act as an innate immune mediator, and it also contributes to the induction of MHC class I which is required for the priming of anti-viral cytotoxic T lymphocyte reactions.

## Results

We have reported that corneal endothelial cells induce strong IFN responses to HSV-1 infection^3^. To determine the molecular mechanisms for the IFN induction, we first conducted a network analysis using global transcriptional responses of corneal endothelial cells and searched for pivotal signaling molecules. Using Ingenuity pathway analysis (IPA), the HSV-1-induced network was annotated as antimicrobial responses and inflammatory responses and were characterized as IFN- and NF-κB-related inflammatory responses as well as antigen presentation. The primary transcriptional network function was associated with the IFN responses.

**Figure 1:**
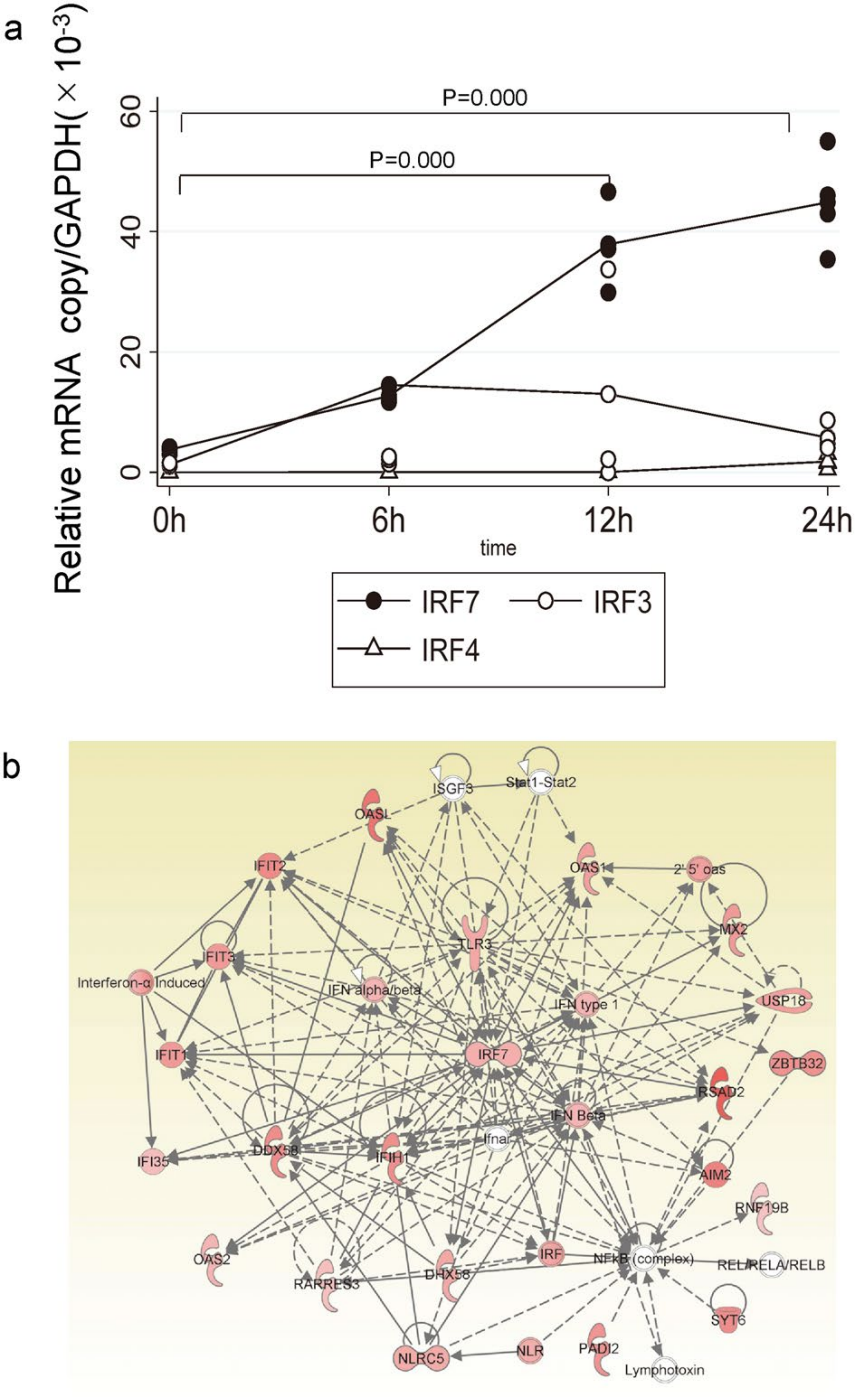
Induction of the expression of interferon regulatory factor 7 (IRF7) in corneal endothelial cells after herpes simplex virus 1 (HSV-1) infection. (**a**) The induction of the mRNA of IRF in human corneal endothelial (HCEn) cells after HSV-1 infection at multiplicity of infection (MOI) 1. IRF7 was induced 6 h post infection (PI), and a significant induction of IRF7 was maintained for 24 h PI. N = 4, ANOVA and Tukey test. (**b**) Network analysis of corneal endothelial cells after HSV-1 infection. IRF7 is centrally located in the highly significant primary network (*P* = 10^–36^). Ingenuity pathway analysis 2020 (QIAGEN Inc. accessed on July 2020, https://digitalinsights.qiagen.com/products-overview/discovery-insights-portfolio/analysis-and-visualization/qiagen-ipa/) was used to generate the network map.

IRF3, IRF4, and IRF7 of the IRF family are generally known to play roles in the induction of type I IFN. However, it has not been determined whether corneal endothelial cells can induce these transcriptional factors. To determine this, we first examined whether the IRFs are induced by human corneal endothelial cells (HCEn) after HSV-1 infection by real-time RT-PCR (Fig. 1a). Of the IRFs, the mRNAs of IRF3 and IRF7 were significantly induced at 6 h post infection (PI). The IRF7 induction was prolonged, and the level was still increased at 24 h.

We next analyzed the transcriptional network of HCEn cells after HSV-1 infection and examined the upstream regulators. The four highest significant upstream regulators were *IFN-α* (*P* = 1.7 × 10^–50^), *IRF7* (*P* = 8.9 × 10^–44^), *IFNA2* (*P* = 6.9 × 10^–43^), and *IFNL1* (*P* = 2.0 × 10^–42^). Thus, IFNs and IRF7 were the most significant regulators. Moreover, IRF7 was positioned in the center of the primary network (*P* = 1 × 10^–36^; Fig. 1b).

We then examined how IRF7 was involved in the IFN-β and the inflammatory responses (see Sup. Fig. S1). When IRF7 was inhibited by siRNA transfection, the HSV-1 induced-IFN-β was significantly reduced (Sup. Fig. S1). We then examined whether IRF7 was involved in the type 3 IFN induction. SiRNA blockade of IRF7 significantly reduced the induction of IL-28 at multiplicity of infection (MOI) of 1. In addition, no significant effect was observed for IL-29. The siRNA blockage of IRF7 also reduced the induction of IL-2, however no effect was observed for the inductions of IL-6 or IL-10. We concluded that IRF7 was involved in the IFN responses.

To analyze the roles played by IRF7 in HSV-1 infected corneal endothelial cells in more detail, the genome of the IRF7 gene DNA binding domain (DBD) was disrupted using the CRISPR/CAS9 system. For this, HCEn cells were co-transfected with the IRF7-targeting donor vector and the Cas9 expression vector, and they were cloned using puromycin selection as IRF7 ∆DBD HCEn cells.

**Figure 2:**
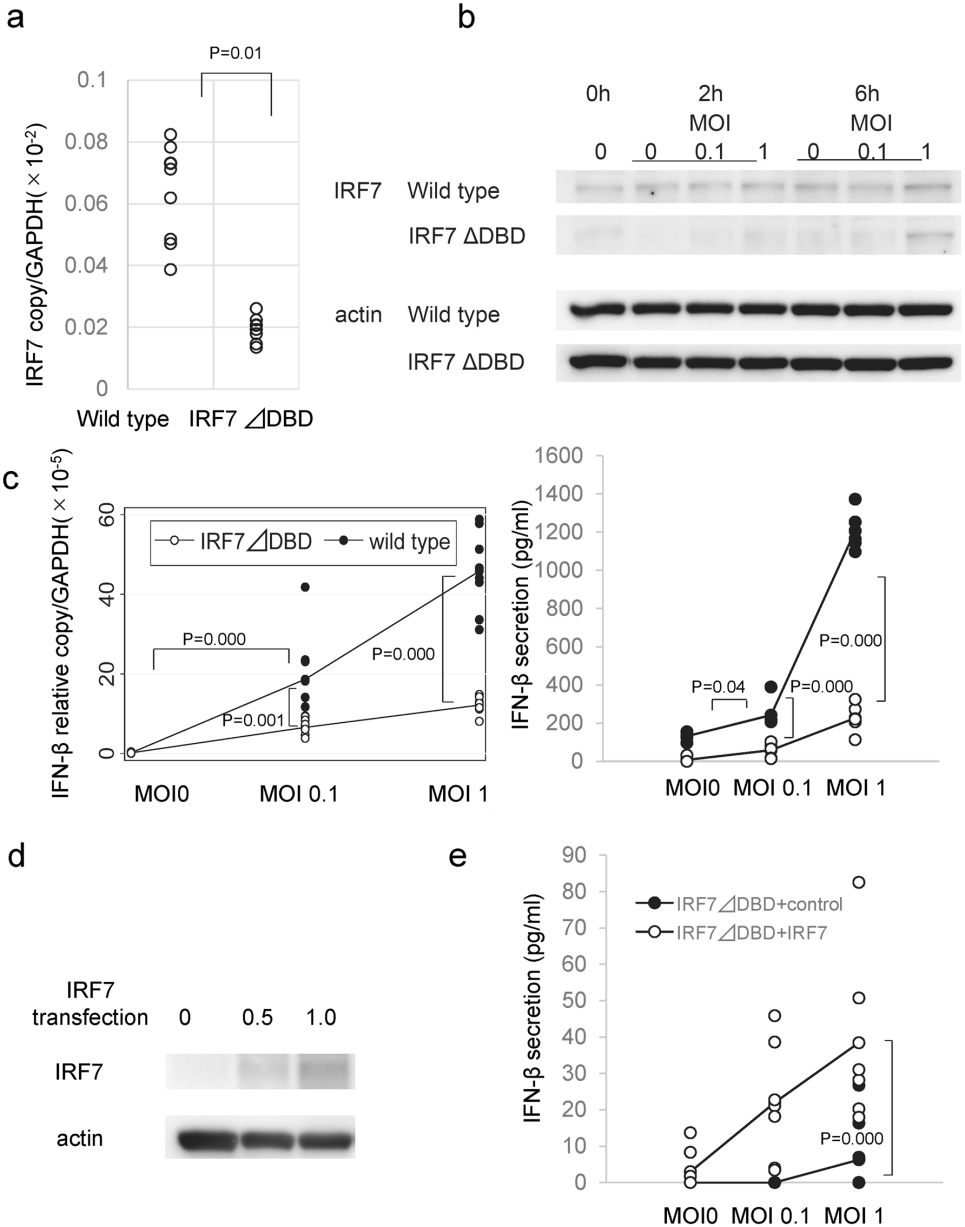
Role of IRF7 in HSV-1-infection-induced interferon β (IFN-β) secretion by the corneal endothelial cells. (**a**) The IRF7 gene DNA binding domain (DBD) of HCEn cells was disrupted by the CRISPR/CAS9 system which then established as IRF7 ∆DBD HCEn cells. The induction of the mRNA of IRF7 was significantly reduced in the IRF7 ∆DBD cells compared to the wild type at 12 h PI at MOI 1. N = 8, ANOVA and Tukey test. (**b**) Induction of IRF7 protein after HSV-1 infection was determined by Western blot. IRF7 protein expression is reduced in IRF7 ∆DBD cells. (**c**) The HSV-1 infection-induced IFN-β induction and secretion are significantly reduced in IRF7 ∆DBD cells at 12 h PI. N = 6, ANOVA Tukey test. (**d**) IRF7 ∆DBD HCEn cells were transfected with an IRF7 expression plasmid and assessed for the expression of the IRF7 protein by western blot. (**e**) The impaired IFN-β secretion in IRF7 ∆DBD HCEn cells is restored by transfection of IRF7 expression plasmid at 12 h PI. N = 8, ANOVA Tukey test.

The efficacy of the IRF7 disruption on the IRF7 ∆DBD HCEn cells was assessed by real-time RT PCR and western blotting (Fig. 2). HSV-1 infection significantly induced the expression of the mRNA of IRF7 at 12 h PI (Fig. 2a). This induction was significantly reduced in IRF7 ∆DBD HCEn cells. Western blot analysis showed that HSV-1 infection induced IRF7 protein at 6 h PI, (Fig. 2b) and this expression was significantly reduced in IRF7 ∆DBD HCEn cells.

After an HSV-1 infection, the mRNA of IFN-β is induced transcriptionally, and a high amount of IFN-β was secreted after 12 h PI (Fig. 2c). This IFN-β induction was significantly impaired in IRF7 ∆DBD HCEn cells (Fig. 2c).

To confirm that IFN-β secretion was mediated by IRF7, IRF7 ∆DBD HCEn cells were transfected with IRF7 DNA. The IRF7 transfection rescued the expression of IRF7 protein (Fig. 2d). To confirm that IFN-β induction was mediated by IRF7, IRF7 ∆DBD HCEn cells transfected with IRF7 DNA were stimulated by HSV-1 infection and assayed for IFN-β secretion. The IRF7 DNA transfection significantly increased the HSV-1-induced-IFN-β secretion (Fig. 2e).

**Figure 3:**
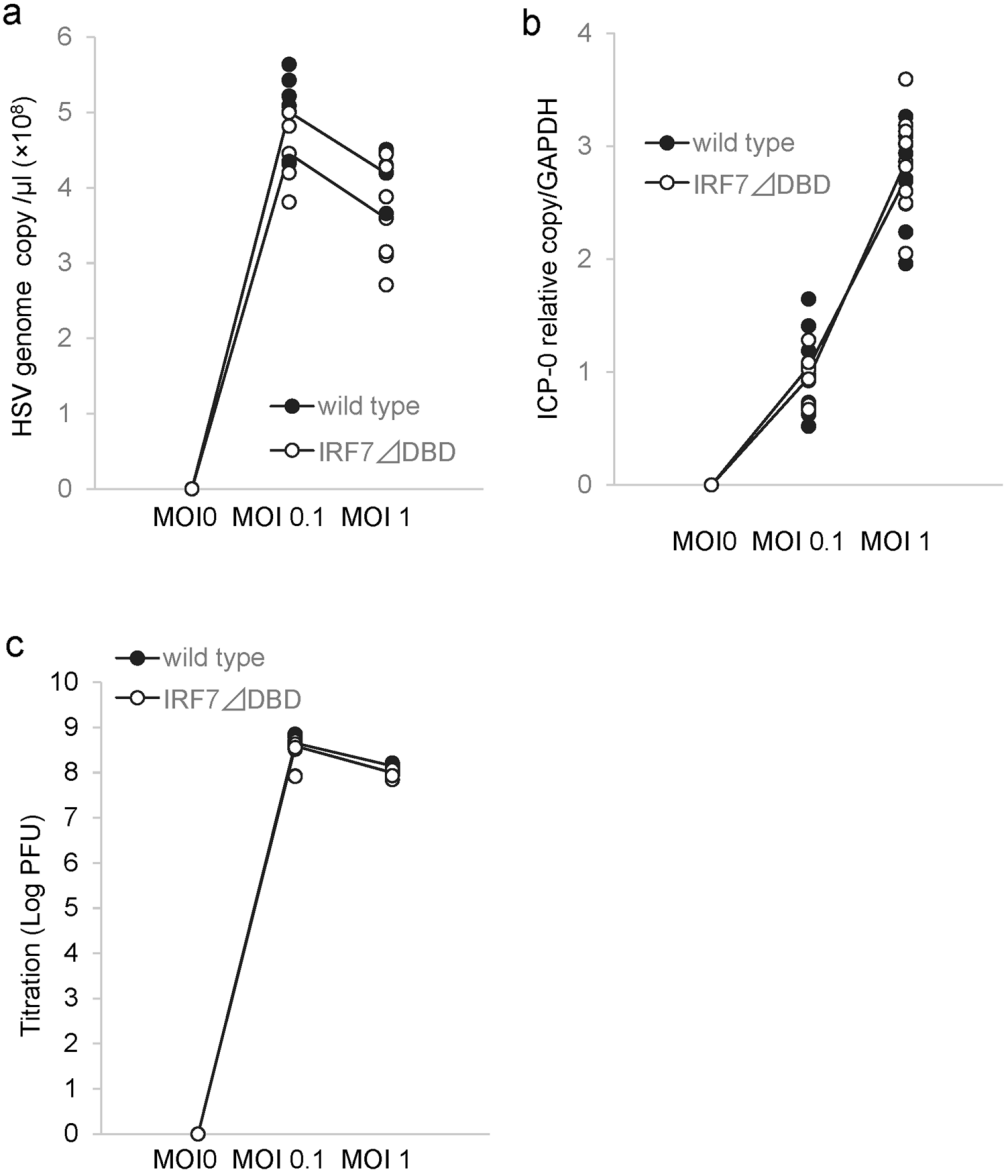
Unperturbed viral replication by IRF7 in corneal endothelial cells. (**a**) The level of HSV-1 DNA in HCEn cells was determined at 24 h after the infection using real-time PCR. The amount of HSV-1 DNA is not reduced for IRF7-competent wild type HCEn cells. N = 6. (**b**) Transcript of an immediately early gene infected cell protein 0 (*ICP0*) of HSV-1, was evaluated in HCEn cells at 12 h after infection using real-time RT-PCR. ICP-0 induction is not perturbed for IRF7-competent wild type HCEn cells. N = 8. (**c**) Titration plaque assay for HCEn cells after infection. The HSV-1 titer is not perturbed for IRF7-competent wild type HCEn cells. N = 6.

IRF proteins are known to exert strong antiviral responses and inhibit viral proliferation. Therefore, we assessed whether IRF7 will reduce viral replication in HCEn cells after HSV-1 infection using real-time PCR. After HSV-1 infection, HSV-1 replicated in the HCEn cells, and the level of the viral genome significantly increased. Interestingly, the viral replication of IRF7 ∆DBD HCEn cells was not affected (Fig. 3a). We next assessed whether IRF7 will play any roles in the transcription of the immediate early gene induction by HSV-1. When ICP0 induction of HSV-1 was assessed by real-time RT PCR, similar induction of ICP0 was observed for IRF7 ∆DBD HCEn cells as that of wild type cells (Fig. 3b). We further assessed whether IRF7 played any role in inhibiting viral proliferation using titration plaque assay. Again, no appreciable effect was observed for IRF7 ∆DBD (Fig. 3c). These findings indicated that IRF7 is not able to directly suppress viral replication after HSV-1 infection.

Effective layer for viral protection is the priming of the anti-viral CD8 T cells responses. Type I interferon induction is indispensable for the reinforcement of anti-viral CD8 responses by antigen presenting cells^6,7^. HSV-1 specific CD8 + T lymphocytes recognize viral peptides in an MHC class I restricted manner, and they generally recognize UL37 as a representative HSV-1 epitope^8^.

**Figure 4:**
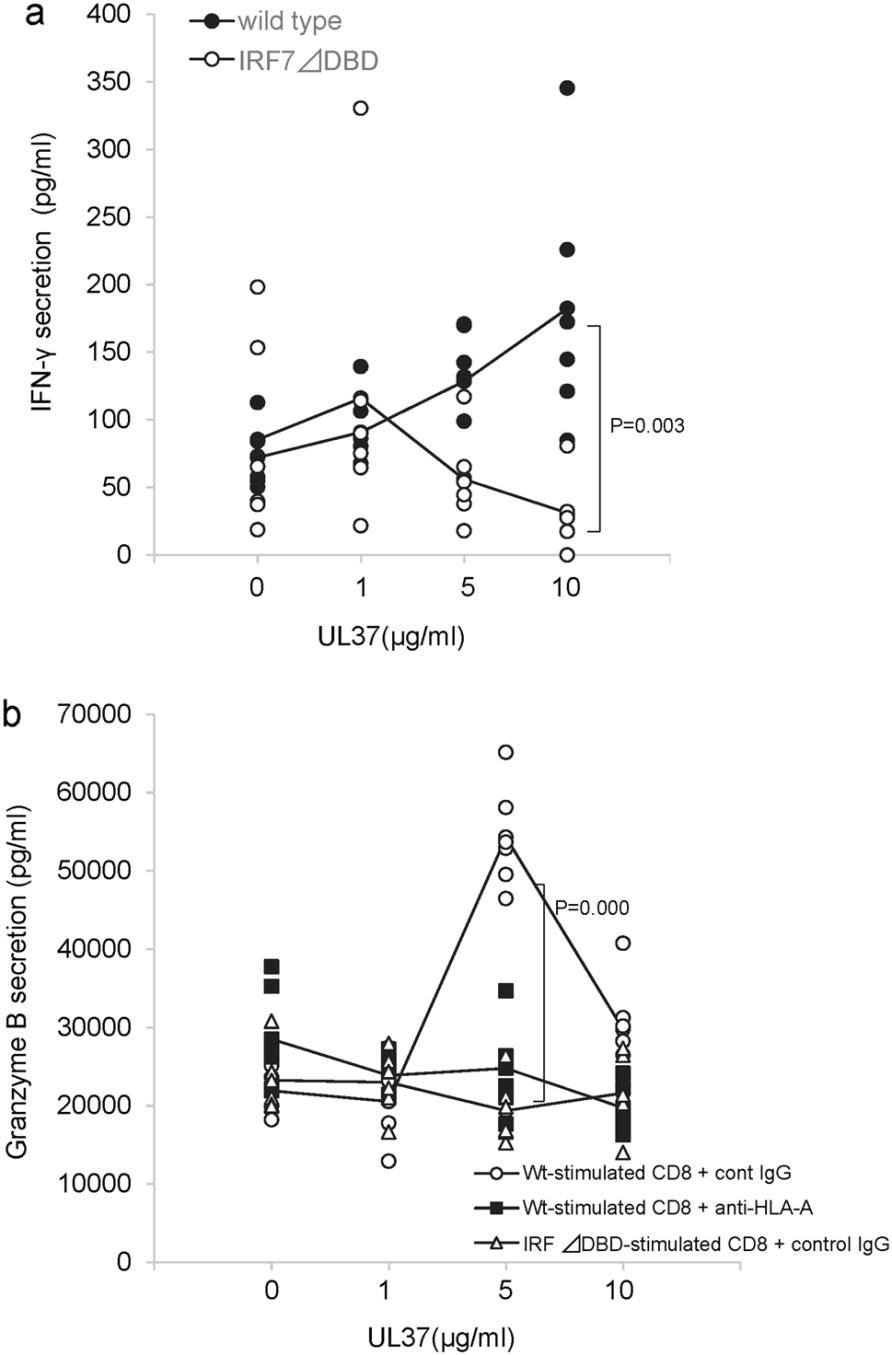
Role of IRF7 for CD8 cytotoxic T lymphocyte priming by infected HCEn cells. (**a**) Peripheral blood-derived allo-CD8 + T lymphocytes from MHC class I matched donor (A*2402) were stimulated by HSV-1-infected HCEn cells. The expanded CD8 + lymphocytes after 2 weeks were re-stimulated with UL37 epitope peptide-pulsed HCEn cells. The supernatant collected 6 h after stimulation was assessed for IFN-γ and granzyme B secretion. IFN-γ secretion of CD8 memory T cells is abolished for stimulation by IRF7 ∆DBD HCEn. N = 6, ANOVA Tukey test. (**b**) HCEn-stimulated CD8 + T cells show viral epitope-induced granzyme B secretion. This granzyme B secretion is abolished by anti-MHC class I antibody. Granzyme B secretion by CD8 memory T cells is blocked for stimulation by IRF7 ∆DBD HCEn. N = 6, ANOVA Tukey test.

Therefore, we evaluated the role of IRF7 in the antigen presentation capability of HCEn cells to combat viral infections. HCEn cells (HLA A2402) were primed with the UL37 epitope peptide and cocultured with CD8 memory T lymphocytes from HSV-1 seropositive HLA A24 donors. CD8 T cells stimulated by HCEn cells significantly secreted IFN-γ and granzyme B as a CTL effector function (Fig. 4a,b). Granzyme B secretion from the cytotoxic CD8 T cells was abolished when CD8 memory T cells were stimulated by IRF7 ∆DBD HCEn cells (HLA A*2402) or by anti-HLA class I blockade (Fig. 4b). IFN-γ secretion was also abolished in IRF7 ∆DBD HCEn cells.

We then assessed how IRF7 contributed to the priming of CD8 memory cells, and focused on the antigen presentation process which was indicated by the network analyses (Fig. 1).

For antigen presentation, viral proteins were processed by proteasome. Processed peptides enter the endoplasmic reticulum (ER) by TAP transporters. MHC class I chain synthesized in the ER are associated with β2 microglobulin. The complete MHC class I were transferred with processed viral peptides and transported to the cell surface.

**Figure 5:**
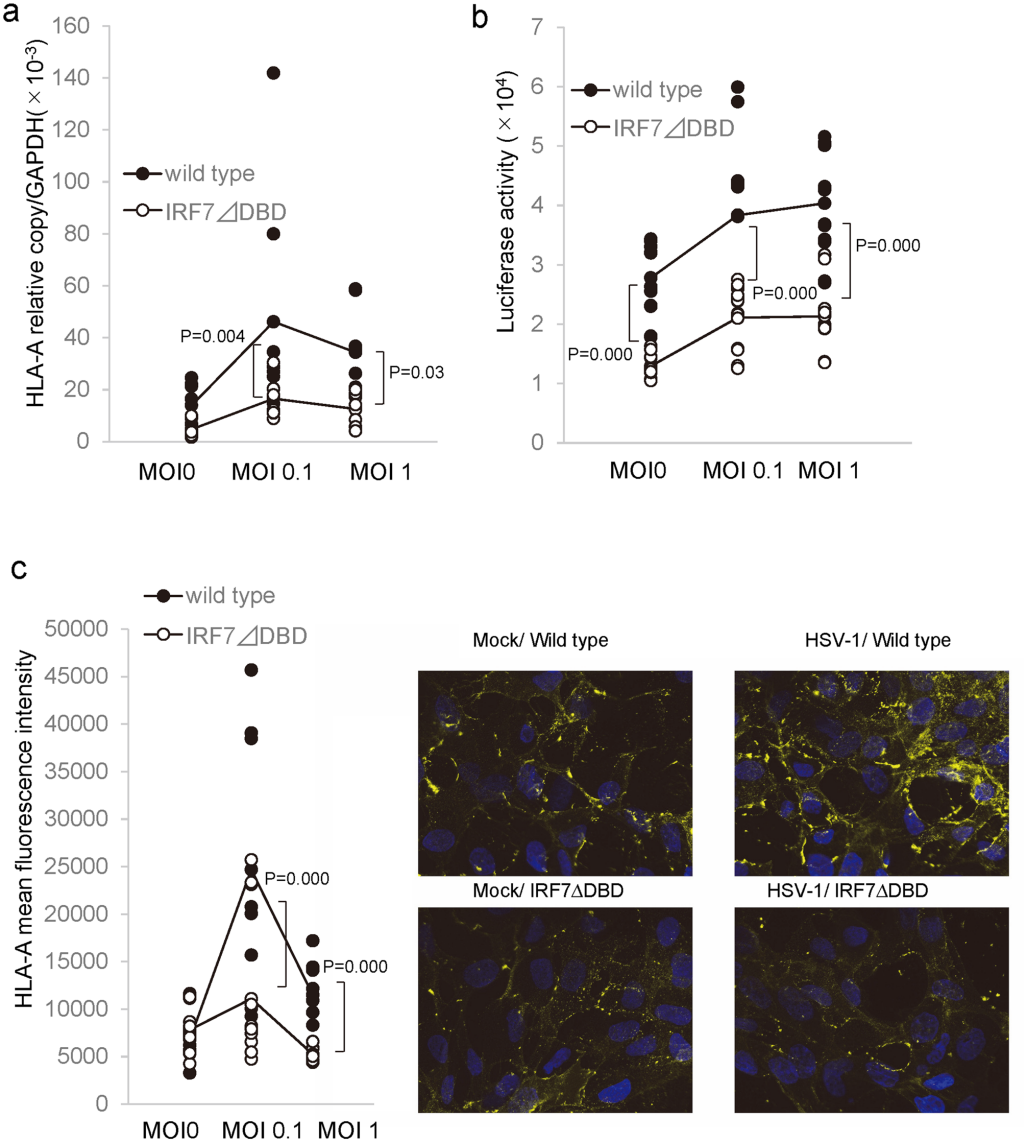
IRF7-dependent MHC class I induction after HSV-1 infection. (**a**) Induction of HLA-A mRNA is significantly reduced in IRF7 ∆DBD cells compared to wild type at 12 h PI. N = 8, ANOVA Tukey test. (**b**) HLA-A2402 promoter reporter vector was transfected into HCEn cells, and the reporter activity after infection was measured using luciferase activity. N = 12, ANOVA and Tukey test. (**c**) Upregulation of HLA-A expression in HCEn cells after HSV-1 infection (12 h PI) by immunohistochemistry (MOI 1). HSV-1-infection-induced HLA-A expression is reduced for IRF7 ∆DBD cells at MOI 0.1 and MOI 1. The signal intensity of HLA-A expression was assessed for 25,000 cells in 14 high powered fields/group. Cells were analyzed using hybrid cell count software (BZ-H4C, Keyence, Osaka, Japan, https://www.keyence.com/ss/products/microscope/bz-casestudy/?tag_tips=Medical%20/%20Life%20Sciences).

We then assessed whether impaired IRF7 can affect the induction of the proteasome 20S subunit (LMP2), TAP1, β2 microglobulin, and MHC class I. These antigen presentation-related molecules in HCEn cells were significantly induced after HSV infection (Sup. Fig. S2). Induction of LMP2 and β2 microglobulin was significantly impaired in the IRF7 ∆DBD cells (Fig. 5a). To examine whether this impaired induction of MHC class I was regulated by MHC class I promotor binding activity, HCEn cells were measured for MHC class I promotor activity. For this, HCEn cells were transfected with a luciferase reporter vector for MHC class I and assayed for luciferase activity (Fig. 5b). Our results showed that HSV-1 infection significantly increased the promoter activity of the wild type HCEn cells. In contrast, IRF7 ∆DBD HCEn cells were impaired for infection-induced promoter activation. To confirm whether this impaired promoter activity can translate into reduced expression of MHC class I, HCEn cells were assessed for surface expression of MHC class I protein. Our results showed that HSV-1 infection stimulated MHC class I expression on the cell surface (Fig. 5c). This infection-induced MHC class I expression was not present in IRF7 ∆DBD cells.

## Discussion

Herpetic and CMV infections are the leading causes of corneal endotheliitis, and they can lead to severe visually depressing bullous keratopathy. These pathogens persist in the cornea and are often reactivated to cause recurrences. To determine how corneal endothelial cells cope with this assault, we analyzed the transcriptional landscape of HSV-1 infected corneal endothelial cells. We have investigated how corneal endothelial cells activate the innate and acquired immune systems.

Earlier, IRF7 was considered to be a plasmacytoid DC specific transcription factor that mediated the production of IFN. Our findings showed that IRF7 is significantly induced in the corneal endothelial cells after HSV-1 infection, and they induced a strong type 1 IFN response as an innate immune system.

Our results showed that IRF7 specifically mediated the infection-induced MHC class I upregulation which was not recognized previously. Furthermore, IRF7 was required for the anti-viral cytotoxic CD8^+^ T cell response which is an effective strategy to suppress viral infections^9^.

To protect the cornea from HSV-1 infections, the first line defense is the immediate induction of type 1 IFN response. When endothelial cells are exposed to HSV-1, a type I IFN response induces ISGs to eradicate the viral pathogens. This IFN response and ISG induction are part of the innate immune system and are immediately activated by pathogen-associated molecular patterns using pattern recognition receptors including the Toll-like receptors (TLRs) and NOD-like receptors. This is an immediate and effective strategy to protect the cells from viral pathogens. In the primary network (Fig. 1a), a number of anti-viral molecules or receptors including *NLR, OASL, RSAD2, AIM2, DHX58, DDX58, RARRES3, OAS2, TLR3*, or *MX2*, are associated. This may explain the redundancy of the anti-viral responses when interferon responses are impaired.

Nevertheless, the IFN response is often breached by HSV-1. This necessitates an alternate and more robust strategy which can eradicate the viral pathogens in a precise and specific manner. This can be typically achieved by the CD8 T lymphocyte arm of the acquired immune system. This direct and precise killing of infected cells or pathogens is mediated by a CD8 cytotoxic T lymphocyte reaction. To initiate this, processed viral epitopes need to be bound to MHC class I for presentation. In corneal endothelial cells, the antigen processing machinery include the MHC class I, LMP2, TAP1, and β2 microglobulin that are upregulated after a viral infection.

To understand how IRF7 promotes the expression of MHC class I, we examined the promoter regulation of MHC class I A*2402. This allele is shared by a high percentage of the general population, and it possesses a conserved ISRE motif on its promoter. ISRE is conserved among the promotors in the HLA class IA, 1B, and 1C, and they serve as putative binding sites for the IRF proteins^10^. Using a class I promoter reporter assay, we showed that IRF7 is required for the transcriptional regulation of MHC class I upregulation.

Thus, corneal endothelial cells have this APC capability and prime CTL reactions^4^. Importantly, IRF7 was required for MHC class I induction and priming of the CTL cells, although some additional pathway, including IL-6, NLRC5, and STAT will be required for MHC class I induction machinery^11,12^.

This mode of protection has two advantages as an anti-viral strategy. First, reactivated viral pathogens are immediately responded to by the activation of the innate immune system and CD8 because the reactivation occurs in the same latently infected cells. Second, no new recruitment of professional APC is required. The endothelial cell-lined anterior chamber is a well-known immune privileged site and is devoid of vasculature with only a few antigen presenting cells. Moreover, strong inflammatory responses to recruit antigen presenting cells is undesirable to maintain corneal clarity.

IRF transcriptional factors exert their roles by binding to consensus ISRE regions by their DNA binding domain. CIITA also possesses ISRE on its promotor, and its induction was reduced in IRF7 ∆DBD HCEn cells. (Sup. Fig. S1) However, the utilization of MHC class II induction is typically characteristics of professional antigen presenting cells and not of non-professional cells including endothelial cells. In addition, the CD4 arm of protection is known to be less effective and can become pathogenic such as in herpetic stromal keratitis. Thus, the roles of IRF7 appear to focus on type I IFN induction and priming of the CD8 arm.

The IRFs that contribute to anti-viral protection are IRF1, IRF3, IRF5, and IRF7^13,14^. Of these, IRF3 and IRF7 play pivotal roles for early and late IFN responses^15^. For example, upon viral infection, viral DNA or RNA is recognized by the cytosolic and endosomal sensing systems which require IRF3 and IRF7 for type I IFN induction^16^.

However, the use or expression of IRFs is highly cell dependent. IRF3 and IRF7 have distinct expression profiles depending on the cell type. IRF3 is ubiquitously expressed in a number of cell types, while IRF7 expression is characteristic for pDC^17^. IRF7 regulates the innate immune arm of pDC and APCs by stimulating IFN responses. Secreted IFN conditions and stimulates the acquired arm of immunity.

We have reported that corneal endothelial cells have IFN-mediated anti-viral responses and antigen presenting functions as a major transcriptional network after viral infections including HSV and CMV^4,23^. Based on the transcriptional network analysis, we found that IRF7 is similarly positioned in the hub of the IFN response after infections by HSV and CMV. Moreover, IRF7 is induced by type I IFN. This can form a positive feedback loop leading to a sustained expression of IFNs^18–20^. These findings suggest that corneal endothelial cells utilize IRF7 as canonical mediators to protect them from susceptible viral pathogens.

An in vivo study has shown that IRF7 can restrict viral replication^21^. For infections by HSV-1, dengue virus, and lymphocytic choriomeningitis virus, IRF7 plays a critical role in viral protection as was shown in IRF7-deficient mice^22–24^.

In an acute HSV-encephalitis model, an IRF7 deficiency was associated with reduced IFN responses, increased viral titer in the brain, and high mortality^22^. In lymphocytic choriomeningitis virus infections, IRF7 deficient mice had impaired control of viral replication in the brain and reduced serum IFN-α levels. Interestingly, the generation of a viral epitope specific CD8 T cell was not affected. This indicated that IRF7 is dispensable or not required for major antigen presenting cells in the brain.

In humans, homozygous IRF7 deficiency was shown to be associated with severe influenza A virus infection and acute respiratory distress syndrome. The IRF7 deficient pDC was severely impaired for type I or type III IFN production in response to influenza A virus infection^25^. Moreover, an increased viral replication and reduced type I IFN production was observed for IRF7-deficient fibroblast or pulmonary epithelial cells in culture. In marked contrast to earlier observations, our data showed that IRF7 did not play pivotal roles in reducing the viral load in corneal endothelial cells. This suggests that IRF7 has a corneal endothelium specific role, and other IRFs can compensate depending on the tissue^21^.

IFN responses are generally beneficial especially for the earlier phases of viral infections. However, prolonged or delayed IFN responses can become harmful and cause significant damage to the host cells. Early IFN administration protects mice against the Middle East respiratory syndrome–coronavirus (MERS-CoV) infection, however a late administration results in depressed viral clearance and fatal pneumonia^26^. In addition, IFN may promote viral infections. For example, SARS-Cov-2 infection, a viral entry receptor, ACE2, is upregulated by type I IFN and allows effective viral entry^27^.

There are some limitations in our study. HCEn cells are very similar in their cytokine and IFN responses to primary cultured corneal endothelial cells^4,28,29^. Although the HCEn cells may not exactly mirror the physiological responses of primary cultured cells, the difficulty in subculturing primary corneal endothelial cells allows only low passage cells for experimentation. Because of this, gene-editing experiments of primary cells are difficult to execute which affects the reproducibility of the infection experiments or may even cause unexpected experimental biases.

In conclusion, our results showed that HSV-1 infection induces IFN responses, and the corneal endothelial cells use IRF7 as a canonical upstream mediator. The roles played by IRF7 are the viral infection-induced MHC class I upregulation and priming of the CD8 arm of acquired immunity, and not simply limited to the type I IFN response. Because of the difficulty of completely removing the pathogens from the cornea, this information may help develop efficacious immune therapy to cope with refractory viral keratitis.

## Material and methods

### Cells

Human corneal endothelial cell line (HCEn cells, HLA-A2402)^2,28,30^ were propagated to confluence in Dulbecco's modified Eagle's medium (DMEM; Gibco, Grand Island, NY) supplemented with 10% fetal bovine serum.

### IRF7 gene editing of human corneal endothelial cells by CRISPR/Cas9

HCEn cells disrupted for the DNA binding domain of IRF7 were established by CRISPER-associated nuclease 9 (Cas9)-mediated homologous recombination. For disrupting DBD^31^, gene targeting donor vector was constructed based on HR110PA-1 (SBI System Biosciences, Palo Alto, CA). The target sequence of IRF7 was designed as CTGTTCGGAGAGTGGCTCCTTGG which included a protospacer adjacent motif (PAM), NGG.

To construct the donor vector, the 5’ and 3’ homologous sequences adjacent to the double-strand break site of the IRF7 gene were cloned using the following primers.

5’ homology arms;

Forward: GGGGAGCCTGAGAGATGAG.

Reverse: GAGCCACTCTCCGAACAGC.

3’ homology arms;

Forward: CTCCTTGGAGAGATCAGCAG.

Reverse: TCCTGGCTGTGAACCCTTAG.

Both the 5’ and 3’ cloned sequences were inserted into the left and right arms of the vector for homologous recombination.

The Cas9 expression vector was constructed by the insertion of the target sequence with PAM (CTGTTCGGAGAGTGGCTCCTTGG) (SBI System Biosciences).

For the disruption of the IRF7 gene, HCEn cells were co-transfected with the gene targeting donor vector and Cas9 expression vector using GenePORTER 3000 (Genlantis, San Diego, CA). The transfected HCEn cells were selected for puromycin resistance and cloned. The resulting cell line, HCEn ∆DBD, was confirmed for the deletion by PCR.

The extracted proteins were assayed by western blot analysis using the anti-IRF7 antibody (Santa Cruz Biotechnology, Dallas, TX), the phospho-IRF7 antibody (Cell Signaling, Danvers, MA), and the HRP-conjugated secondary antibody (Cell Signaling, Danvers, MA).

### Virus

HSV-1 viral stocks were prepared using Vero cells infected with the KOS strain of HSV-1 (generous gift from Drs. Kozaburo Hayashi). Viral stocks were purified using sucrose density gradient centrifugation. The viral stocks were aliquoted and stored at – 80 °C until use.

The level of IFN-β, granzyme B, and IFN-γ in the supernatant was measured using a commercial ELISA kits for IFN-β (Antigenix America, Huntington Station, NY), IFN-γ (eBioscience, San Diego, CA), and granzyme B (Mabtech, Nacka Strand, Sweden).

### Real-time RT-PCR

Total RNA was isolated from the infected HCEn cells and reverse transcribed using the QuantiTect Reverse Transcription Kit (Qiagen, Hilden, Germany). The cDNAs were amplified and quantified by the LightCycler (Roche, Mannheim, Germany) using the QuantiTect SYBR Green PCR kit and real-time PCR primer pairs (Supplementary Table S1).

### Transfection

HCEn cells were transfected with an IRF7-expressing plasmids using a CMV promoter (pCMV6 backbone, OriGene, Rockville, MD) with gene porter 3000 (Genlantis, San Diego, CA). The extracted proteins were confirmed by western blot analyses using an anti-IRF7 antibody (Santa Cruz Biotechnology, Dallas, TX) and HRP-conjugated secondary antibody (Cell Signaling, Danvers, MA).

For siRNA transfection, HCEn cells were transfected with IRF7 siRNA (Santa Cruz Biotechnology, Dallas, TX) using RNAifect (Qiagen, Hilden, Germany) according to the manufacturer’s instructions.

### Immunohistochemistry

HCEn cells were fixed in 4% paraformaldehyde and stained with a mouse anti-HLA-A antibody (BioLegend, San Diego, CA) and made visible with Alexa 555 conjugated secondary antibody (BioLegend). The cells were examined with an all-in-one fluorescence microscope (BZ-X800, Keyence, Osaka, Japan) and analyzed using hybrid cell count software (BZ-H4C, Keyence, Osaka, Japan, https://www.keyence.com/ss/products/microscope/bz-casestudy/?tag_tips=Medical%20/%20Life%20Sciences).

### Luciferase reporter assay

Promoter lesion (-290—0) of HLA class I A2402 containing Interferon-sensitive response element (ISRE) motif^10^ was cloned into NanoLuc reporter vector (pNL1.2[NlucP], Promega, Madison, Wisconsin) by XhoI/ EcoRV. The HCEn cells were transfected with the HLA A2402 reporter vector using Helix-IN (Oz Biosciences, San Diego, CA) and infected with HSV-1. The luciferase activity was measured using the Nano-Glo Luciferase assay (Promega).

### HSV-specific cytotoxic T lymphocyte assay for corneal endothelial cells

Peripheral blood mononuclear cells were isolated from heparinized blood of HLA-A*2402-positive donors seropositive for HSV-1 using Ficoll density gradient centrifugation. CD8 + T cells were isolated from the mononuclear cells using a magnet beads based negative selection kit (IMag, BD Bioscience, San Jose, CA). To expand the CD8 + T cells, the HCEn cells infected with HSV-1 (KOS strain) at MOI 0.1 for 24 h were irradiated and cocultured with CD8 + T cells in RPMI supplemented with 10% fetal bovine serum and recombinant IL-2 (10 ng/ml) as described^4^.

HLA-A*2402-restricted peptide, AYLPRPVEF, was used for the HSV-1 epitopes of UL37^8^. The peptide-pulsed HCEn cells were co-cultured with the CD8 T cells at CD8 + T cells/HCEn cells ratio of 1/2. The supernatant collected after 6 h were measured for the released of granzyme B using a commercially available ELISA kit (Thermo Fisher, Waltham, MA). Anti-MHC class I antibody, W6/32 (Biolegend, San Diego, CA) or control antibody, were used for the blockade of MHC class I.

All of the procedures used in this study conformed to the tenets of the Declaration of Helsinki, and they were approved by the Institutional Review Board of Tottori University, Tottori, Japan. An informed consent was obtained from all of the participants.

### Network analysis of corneal endothelial cell transcriptome after HSV-1 infection

HCEn cells were infected with HSV-1 (KOS strain) at multiplicity of infection (MOI) 1 and the RNAs were extracted at 12 h post-infection (PI)^2,3,30^. Transcriptome data were obtained using whole human genome microarray (Agilent Technologies, Santa Clara, CA)^23^. To identify canonical pathways and transcriptional networks that were most significant to the data set^23^, the data were analyzed by the Ingenuity Pathway (IPA; 2020, QIAGEN Inc. accessed on July 2020, https://digitalinsights.qiagen.com/products-overview/discovery-insights-portfolio/analysis-and-visualization/qiagen-ipa/).

### Statistical analyses

Data are presented as the means ± standard error of the means (SEMs). The significance of the differences was determined by *t* tests or ANOVA and post hoc tests.

## Supplementary Information

Supplementary Information.

## Data Availability

The datasets analyzed during the current study are available from the corresponding author on reasonable request.

## References

[1] Farooq AV, Shukla D (2011). Corneal latency and transmission of herpes simplex virus-1. Future Virol..

[2] Takeda S (2011). Roles played by toll-like receptor-9 in corneal endothelial cells after herpes simplex virus type 1 infection. Invest. Ophthalmol. Vis. Sci..

[3] Haruki T (2015). Indoleamine 2,3-dioxygenase 1 in corneal endothelial cells limits herpes simplex virus type 1-induced acquired immune response. Br. J. Ophthalmol..

[4] Miyazaki D (2017). Corneal endothelial cells activate innate and acquired arm of anti-viral responses after cytomegalovirus infection. Exp. Eye Res..

[5] Negishi H, Taniguchi T, Yanai H (2018). The interferon (IFN) class of cytokines and the IFN regulatory factor (IRF) transcription factor family. Cold Spring Harb. Perspect. Biol..

[6] Schiavoni G, Mattei F, Gabriele L (2013). Type I interferons as stimulators of DC-mediated cross-priming: Impact on anti-tumor response. Front. Immunol..

[7] Knuschke T (2018). Induction of type I interferons by therapeutic nanoparticle-based vaccination is indispensable to reinforce cytotoxic CD8(+) T cell responses during chronic retroviral infection. Front. Immunol..

[8] Jing L (2012). Cross-presentation and genome-wide screening reveal candidate T cells antigens for a herpes simplex virus type 1 vaccine. J. Clin. Invest..

[9] Conrady CD, Zheng M, Stone DU, Carr DJ (2012). CD8+ T cells suppress viral replication in the cornea but contribute to VEGF-C-induced lymphatic vessel genesis. J. Immunol..

[10] Ramsuran V (2017). Sequence and phylogenetic analysis of the untranslated promoter regions for HLA class I genes. J. Immunol..

[11] Kobayashi KS, van den Elsen PJ (2012). NLRC5: A key regulator of MHC class I-dependent immune responses. Nat. Rev. Immunol..

[12] Brutkiewicz RR (2016). Cell signaling pathways that regulate antigen presentation. J. Immunol..

[13] Honda K, Takaoka A, Taniguchi T (2006). Type I interferon [corrected] gene induction by the interferon regulatory factor family of transcription factors. Immunity.

[14] Odendall C, Kagan JC (2015). The unique regulation and functions of type III interferons in antiviral immunity. Curr. Opin. Virol..

[15] Jefferies CA (2019). Regulating IRFs in IFN driven disease. Front. Immunol..

[16] Thompson MR, Kaminski JJ, Kurt-Jones EA, Fitzgerald KA (2011). Pattern recognition receptors and the innate immune response to viral infection. Viruses.

[17] Au WC, Moore PA, LaFleur DW, Tombal B, Pitha PM (1998). Characterization of the interferon regulatory factor-7 and its potential role in the transcription activation of interferon A genes. J. Biol. Chem..

[18] Civas A, Island ML, Genin P, Morin P, Navarro S (2002). Regulation of virus-induced interferon-A genes. Biochimie.

[19] Osterlund PI, Pietila TE, Veckman V, Kotenko SV, Julkunen I (2007). IFN regulatory factor family members differentially regulate the expression of type III IFN (IFN-lambda) genes. J. Immunol..

[20] Iversen MB, Paludan SR (2010). Mechanisms of type III interferon expression. J. Interferon Cytokine Res..

[21] Andersen LL (2015). Functional IRF3 deficiency in a patient with herpes simplex encephalitis. J. Exp. Med..

[22] Canivet C (2018). Both IRF3 and especially IRF7 play a key role to orchestrate an effective cerebral inflammatory response in a mouse model of herpes simplex virus encephalitis. J. Neurovirol..

[23] Chen HW (2013). The roles of IRF-3 and IRF-7 in innate antiviral immunity against dengue virus. J. Immunol..

[24] Li W, Hofer MJ, Nocon AL, Manders P, Campbell IL (2013). Interferon regulatory factor 7 (IRF7) is required for the optimal initial control but not subsequent clearance of lymphocytic choriomeningitis virus infection in mice. Virology.

[25] Ciancanelli MJ (2015). Infectious disease. Life-threatening influenza and impaired interferon amplification in human IRF7 deficiency. Science.

[26] Channappanavar R (2019). IFN-I response timing relative to virus replication determines MERS coronavirus infection outcomes. J. Clin. Invest..

[27] Ziegler CGK (2020). SARS-CoV-2 receptor ACE2 is an interferon-stimulated gene in human airway epithelial cells and is detected in specific cell subsets across tissues. Cell.

[28] Sugita S (2009). Human corneal endothelial cells expressing programmed death-ligand 1 (PD-L1) suppress PD-1+ T helper 1 cells by a contact-dependent mechanism. Invest. Ophthalmol. Vis. Sci..

[29] Yamada Y, Sugita S, Horie S, Yamagami S, Mochizuki M (2010). Mechanisms of immune suppression for CD8+ T cells by human corneal endothelial cells via membrane-bound TGFbeta. Invest. Ophthalmol. Vis. Sci..

[30] Miyazaki D (2011). Herpes simplex virus type 1-induced transcriptional networks of corneal endothelial cells indicate antigen presentation function. Invest. Ophthalmol. Vis. Sci..

[31] Antonczyk A (2019). Direct inhibition of IRF-dependent transcriptional regulatory mechanisms associated with disease. Front. Immunol..

