# Supplementary material for: Role of interferon regulatory factor 7 in corneal endothelial cells after HSV-1 infection
Source: Sci Rep. 2021 Aug 13;11:16487. doi: 10.1038/s41598-021-95823-9 (PMC8363731; doi:10.1038/s41598-021-95823-9)
Supplement: Supplementary file 1 — Supplementary Information. [file 41598_2021_95823_MOESM1_ESM.pdf]

Supplementary Table 1. List of primer pairs

IRF3	forward	ACCAGCCGTGGACCAAGAG
	reverse	TACCAAGGCCCTGAGGCAC
IRF4	forward	GACAACGCCTTACCCTTCG
	reverse	AGGGGTGGCATCATGTAGTT
	probe	UPL #3(Roche:04 685 008 001)
IRF7	forward	TGGTCCTGGTGAAGCTGGAA
	reverse	GATGTCGTCATAGAGGCTGTTGG
CIITA TypeⅢ	forward	ACGCCCTGCTGGGTCC
	reverse	AACTCCATGGTGGCACACTG
IL-2	forward	AAGAATCCCAAACTCACCAGGAT
	reverse	TCTAGACACTGAAGATGTTTCAGTTCTG
IL-6	forward	GATGAGTACAAAAGTCCTGATCCA
	reverse	CTGCAGCCACTGGTTCTGT
	probe	UPL #40(Roche:04 687 990 001)
IL-28	forward	AGGGCCAAAGATGCCTTAGA
	reverse	TCCAGAACCTTCAGCGTCAG
IL-29	forward	GGACGCCTTGGAAGAGTCAC

	reverse	AGCTGGGAGAGGATGTGGT
ICP0	forward	AGCGAGTACCCGCCGGCCTG
	reverse	CAGGTCTCGGTCGCAGGGAAAC
β2-microglobulin	forward	TGCTGTCTCCATGTTTGATGTATCT
	reverse	TCTCTGCTCCCCACCTCTAAGT
LMP2	forward	CGTTGTGATGGGTTCTGATTCC
	reverse	GACAGCTTGTCAAACACTCGGTT
HLA-A	forward	AAAAGGAGGGAGTTACACTCAGG
	reverse	GCTGTGAGGGACACATCAGAG
TAP1	forward	AGGTACTGCTCTCCATCTAC
	reverse	AGTGTAAGGGAGTCAACAGA
IFN-β	forward	CATTACCTGAAGGCCAAGGA
	reverse	CAATTGTCCAGTCCCAGAGG
GAPDH	forward	AGCCACATCGCTCAGACAC
	reverse	GCCCAATACGACCAAATCC

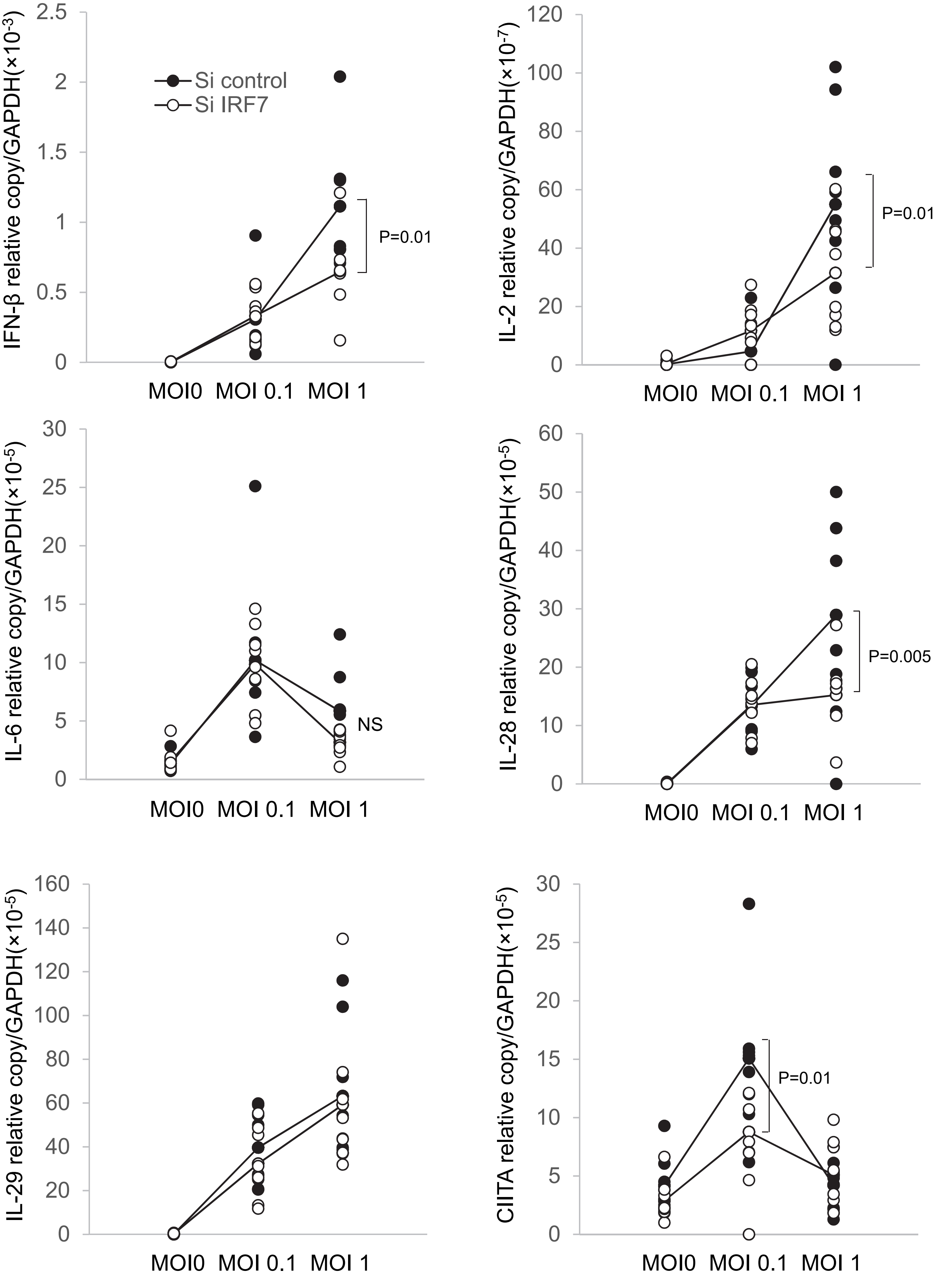

Supplementary Figure 1

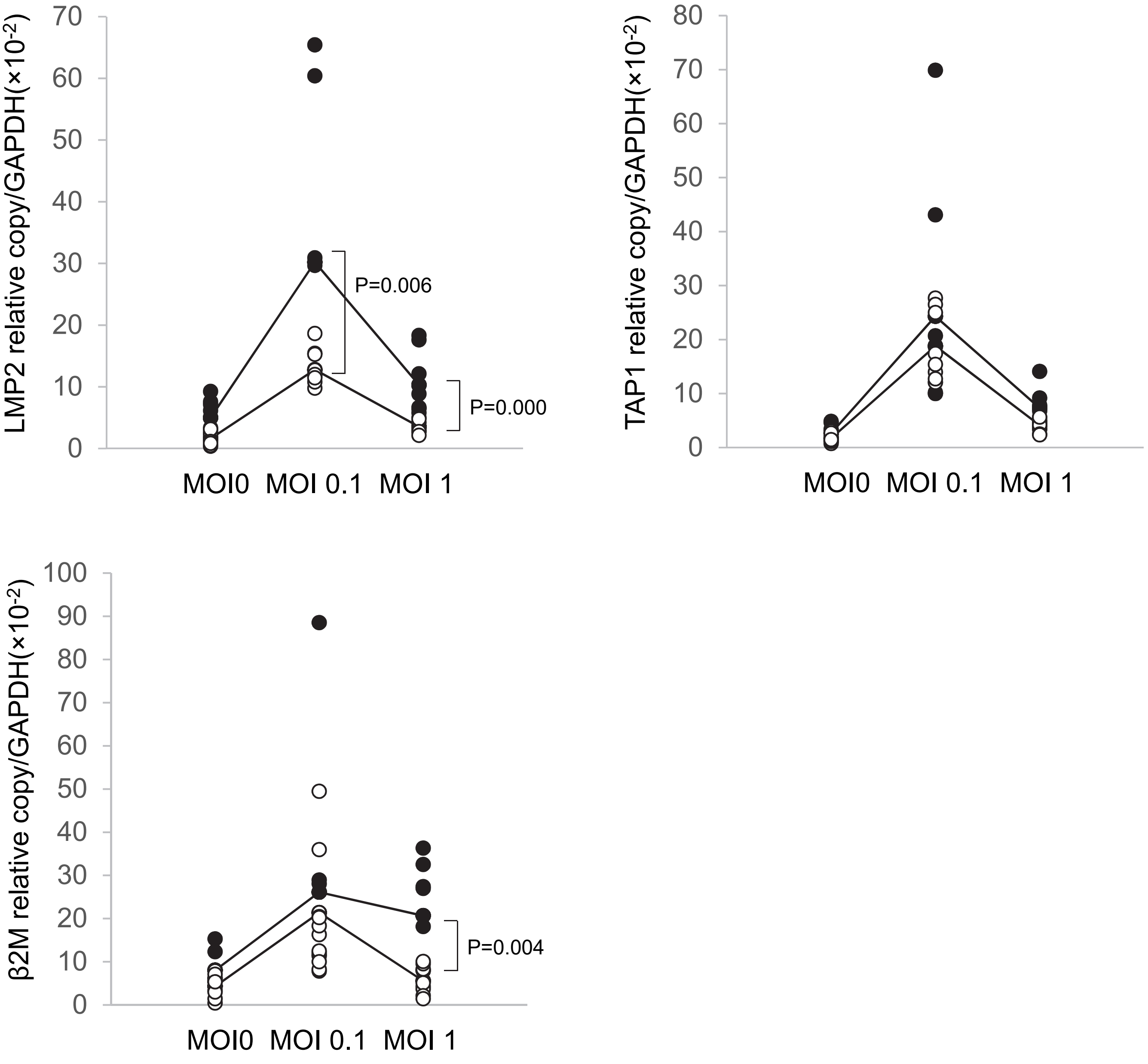

Supplementary Figure 2

Supplementary Figure 1. Effect of IRF7 inhibition on HSV-1-induced cytokine mRNA panel. HCEn cells were transfected with siRNA of IRF7 or control siRNA and infected with HSV-1. Cytokine mRNA induction was assessed using real-time RT-PCR at 12 h PI. The induction of IFN-β and CIITA is significantly reduced by IRF7 inhibition. N = 8, ANOVA Dunnett's test.

Supplementary Figure 2. Effect of IRF7 inhibition on mRNA panel of MHC class I antigen presentation. The expression of the mRNA of LMP2, TAP1, and β2-microglobulin (β2M) was assessed using real-time RT-PCR at 12 h PI. The mRNA of LMP2 induction was significantly reduced for IRF7 ∆DBD HCEn. N = 8, ANOVA Dunnett's test.

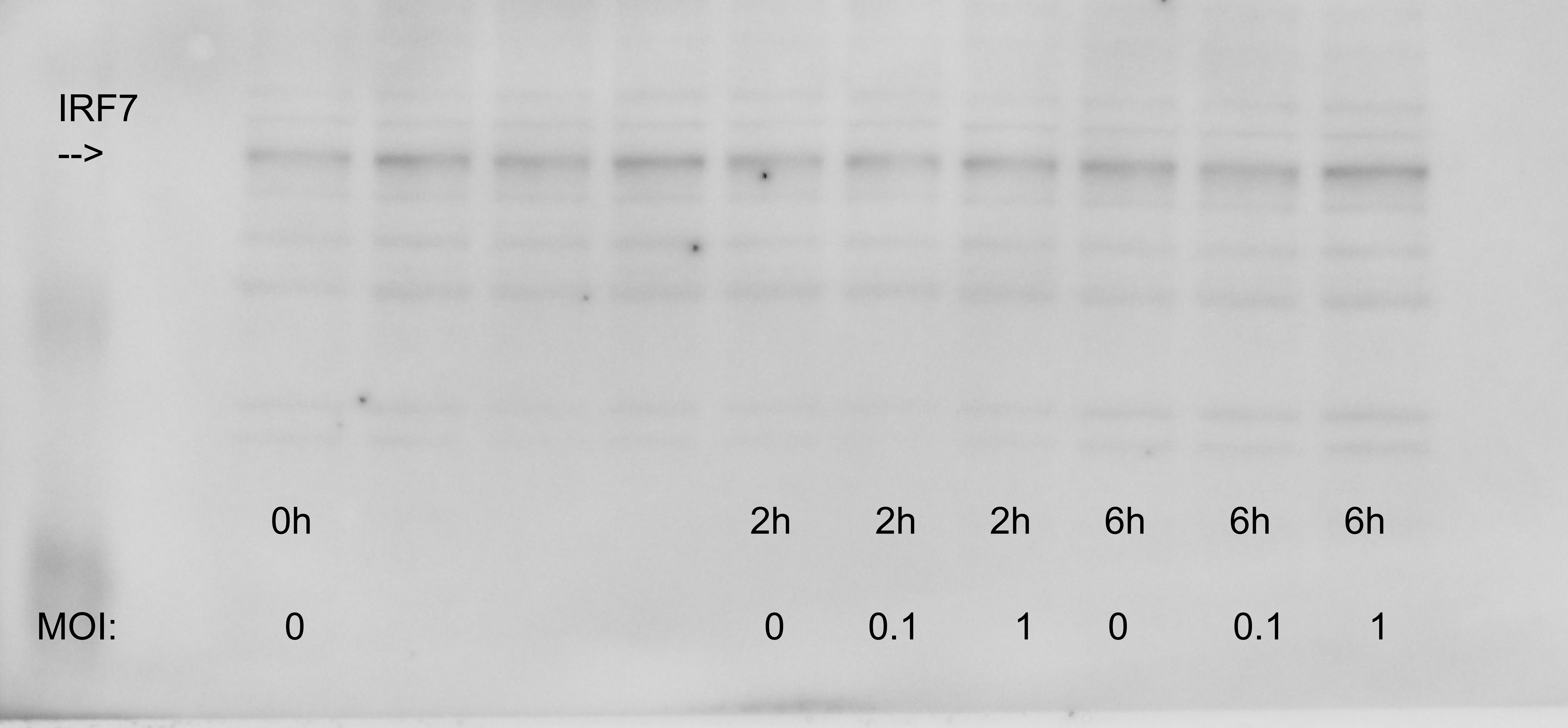

Figure 2b Wild type

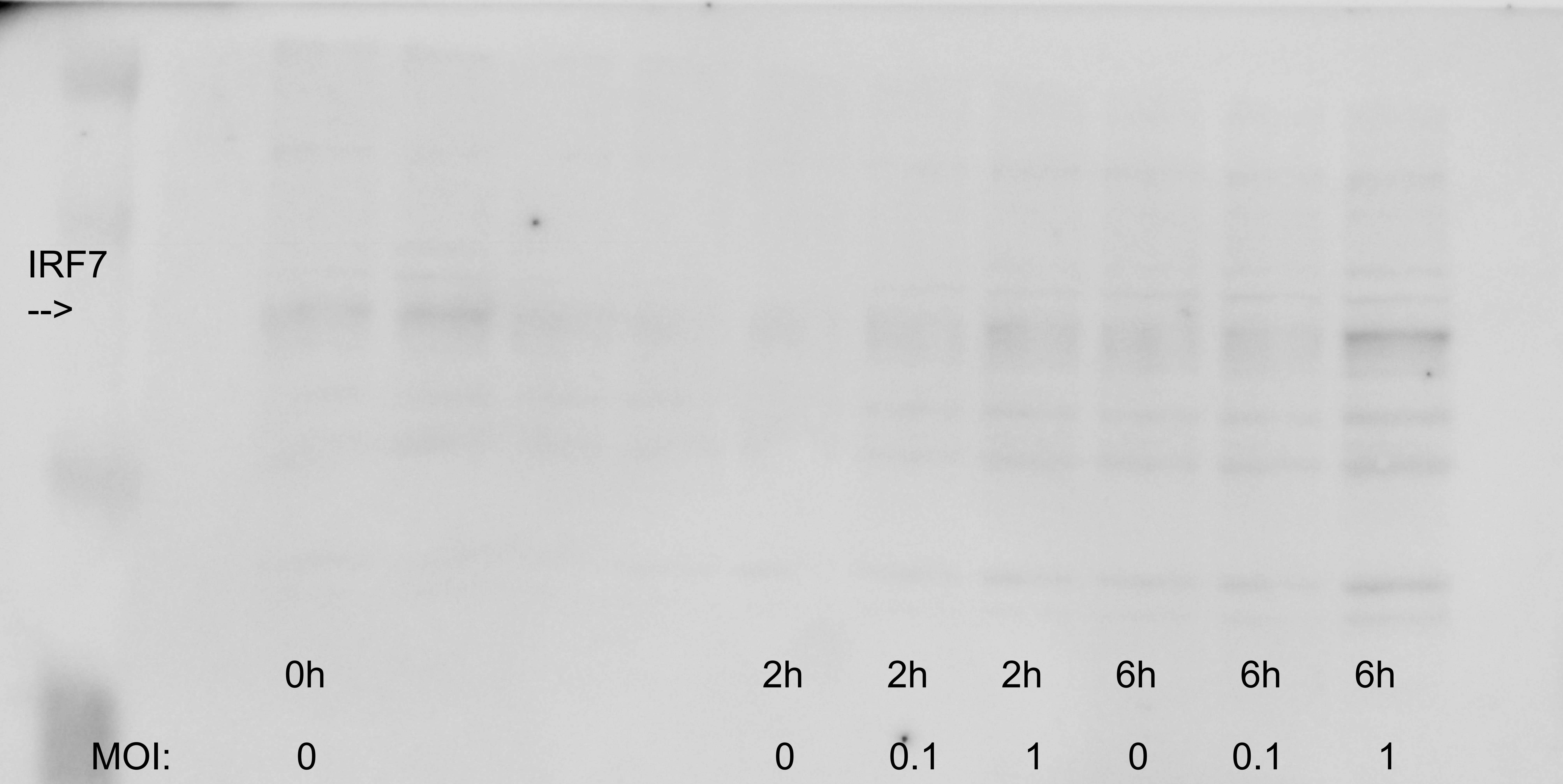

Figure 2b IRF7 ΔDBD

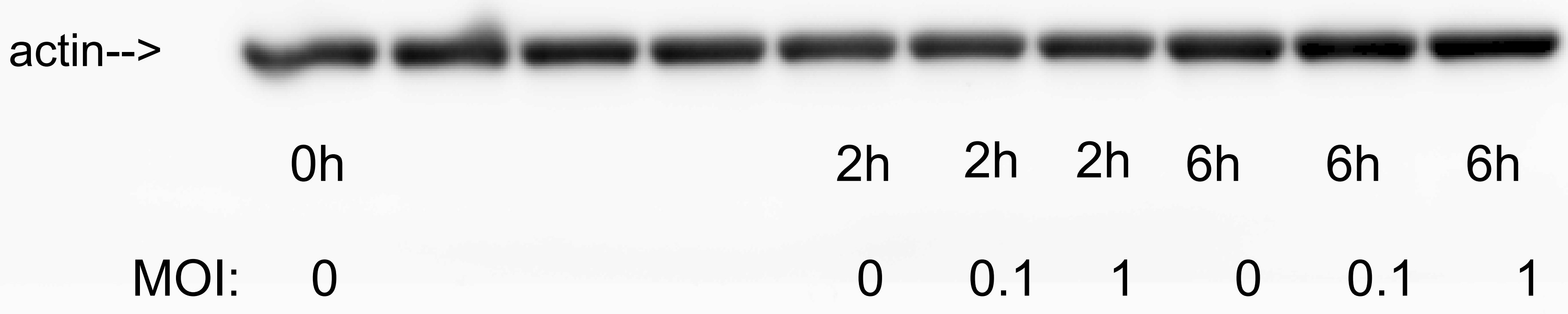

Figure 2b IRF7 wild type

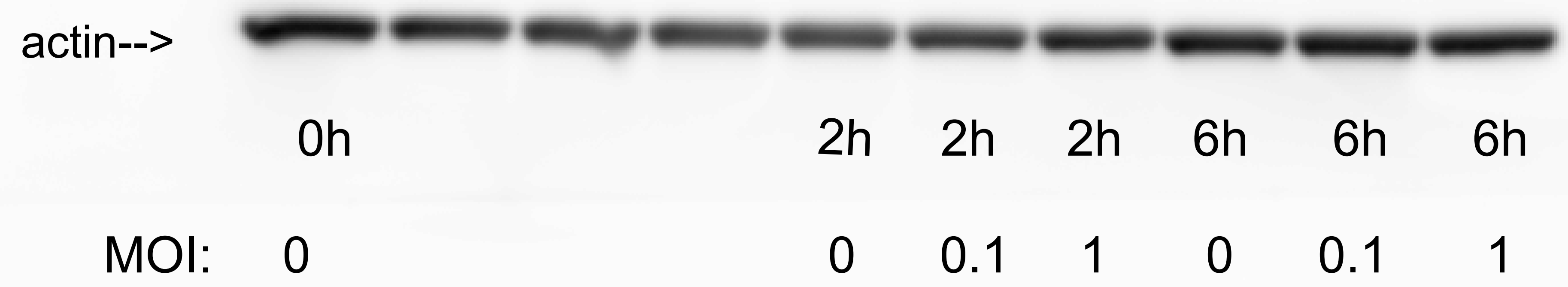

Figure 2b IRF7 ΔDBD

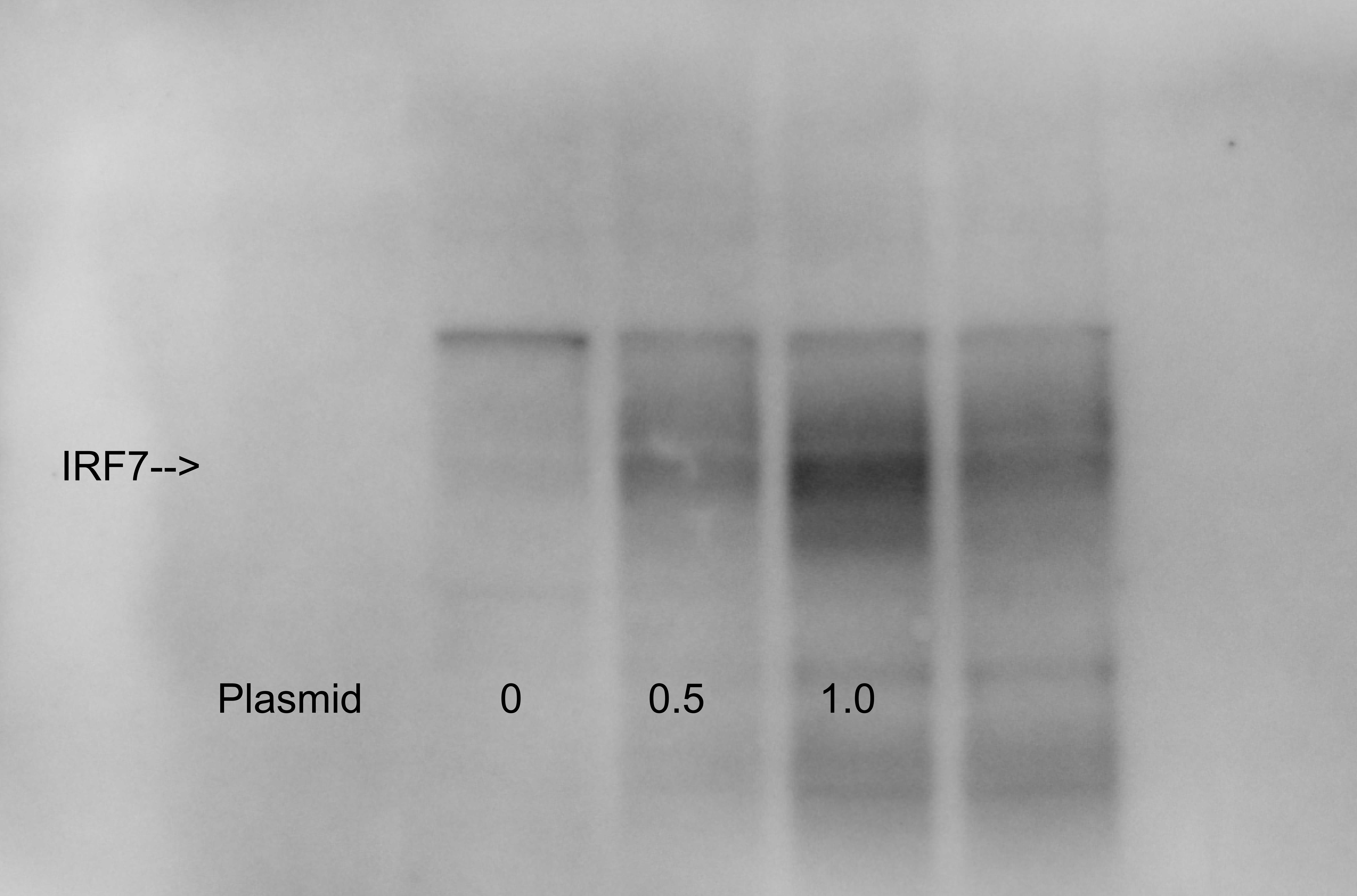

Figure 2d

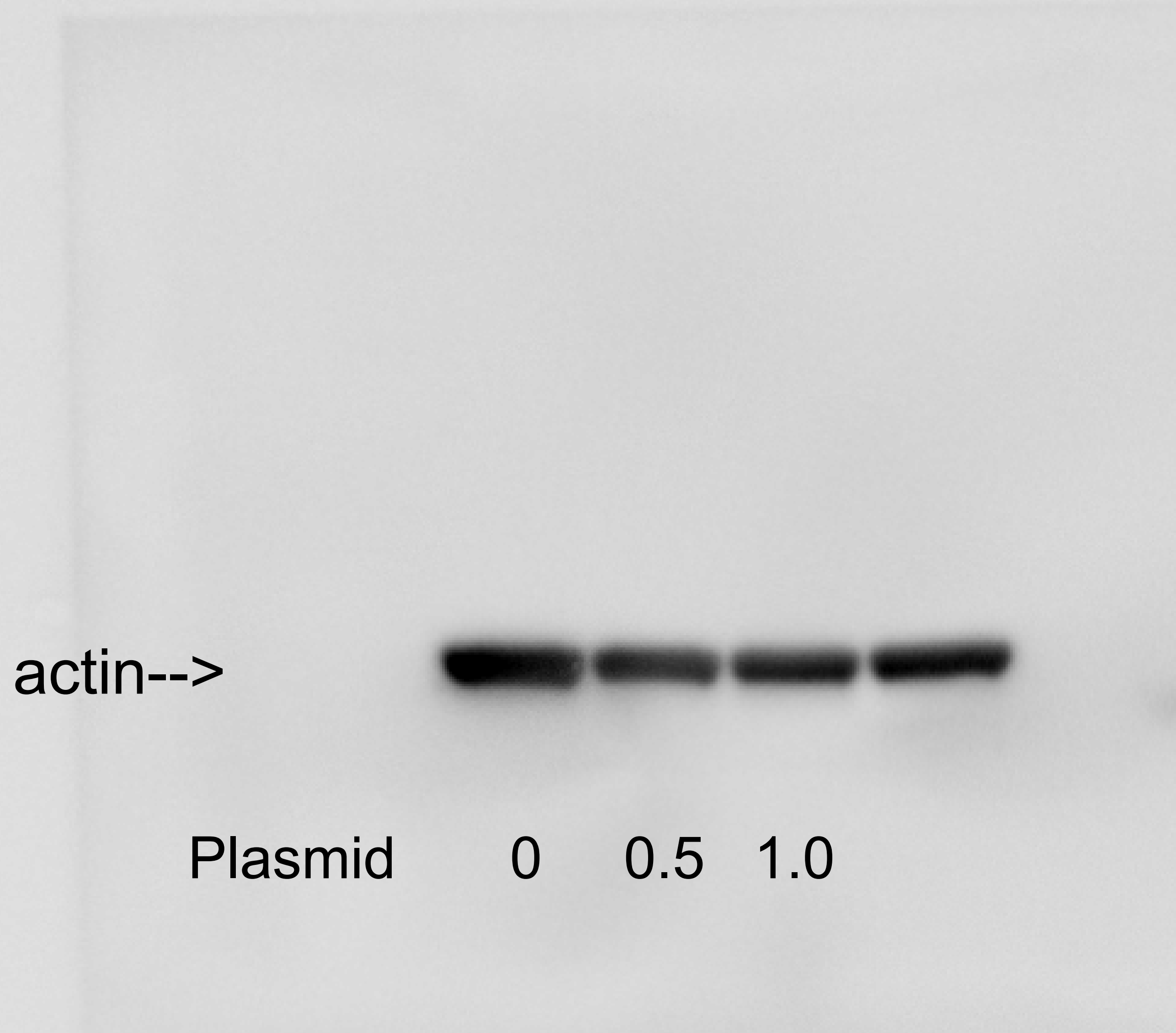

Figure 2d
